# Western Diet Dampens T Regulatory Cell Function to Fuel Hepatic Inflammation in Metabolic Dysfunction-Associated Steatotic Liver Disease

**DOI:** 10.3390/cells15020165

**Published:** 2026-01-16

**Authors:** Sudrishti Chaudhary, Ravi Rai, Pabitra B. Pal, Dana Tedesco, Daniel Rossmiller, Biki Gupta, Aatur D. Singhi, Satdarshan P. Monga, Arash Grakoui, Smita S. Iyer, Reben Raeman

**Affiliations:** 1 Pittsburgh Liver Research Center, School of Medicine, University of Pittsburgh, Pittsburgh, PA 15213, USA; schudhry@pitt.edu (S.C.); raviprakash.rai@pitt.edu (R.R.); pbp13@pitt.edu (P.B.P.); guptab45@stanford.edu (B.G.); ads130@pitt.edu (A.D.S.); smonga@pitt.edu (S.P.M.); 2 Division of Gastroenterology, Hepatology and Nutrition, Department of Medicine, School of Medicine, University of Pittsburgh, Pittsburgh, PA 15213, USA; 3 Organ Pathobiology and Therapeutics Institute, Department of Pharmacology and Chemical Biology, University of Pittsburgh, Pittsburgh, PA 15213, USA; 4 Division of Experimental Pathology, Department of Pathology, University of Pittsburgh, Pittsburgh, PA 15213, USA; dar255@pitt.edu (D.R.); siyer.3@pitt.edu (S.S.I.); 5 Emory Vaccine Center, Department of Medicine, School of Medicine, Emory University, Atlanta, GA 30322, USA; danactedesco@gmail.com (D.T.); arash.grakoui@emory.edu (A.G.); 6 Division of Microbiology and Immunology, Department of Medicine, School of Medicine, Emory University, Atlanta, GA 30322, USA; 7 Division of Anatomic Pathology, Department of Pathology, School of Medicine, University of Pittsburgh, Pittsburgh, PA 15213, USA; 8 Division of Infectious Diseases, Department of Medicine, Emory University, Atlanta, GA 30322, USA; 9 Yerkes National Primate Research Center, Atlanta, GA 30322, USA

**Keywords:** MASLD, MASH, fibrosis, inflammation, T regulatory cells

## Abstract

The immunosuppressive T regulatory cells (Tregs) regulate immune responses and maintain immune homeostasis, yet their functions in metabolic dysfunction-associated steatotic liver disease (MASLD) remain controversial. Here we report increased accumulation of Tregs and effector T cells within the liver parenchyma of mice fed a Western diet (WD). This pattern was also observed in MASH patients, where an increase in intrahepatic Tregs was noted. In the absence of adaptive immune cells in *Rag1* KO mice, WD promoted accumulation of intrahepatic neutrophils and macrophages and exacerbated hepatic inflammation and fibrosis. Similarly, targeted Treg depletion exacerbated WD-induced hepatic inflammation and fibrosis. In Treg-depleted mice, hepatic injury was associated with increased accumulation of neutrophils, macrophages, and activated T cells in the liver. Conversely, induction of Treg numbers using recombinant IL2/αIL2 mAb cocktail reduced hepatic steatosis, inflammation, and fibrosis in WD-fed mice. Analysis of intrahepatic Tregs from WD-fed mice revealed a phenotypic signature of impaired Treg function in MASLD. Ex vivo functional studies showed that glucose and palmitate, but not fructose, impaired the immunosuppressive ability of Treg cells. The findings indicate that the liver microenvironment in MASLD impairs the ability of Tregs to suppress effector immune cell activation, thus perpetuating chronic inflammation and driving MASLD progression.

## 1. Introduction

Metabolic dysfunction-associated steatotic liver disease (MASLD) is a progressive form of inflammatory liver disease encompassing a spectrum of liver pathology ranging from simple steatosis to steatohepatitis (MASH, metabolic dysfunction-associated steatohepatitis) [1]. MASLD affects one-third of the Western population, and nearly 20% of the people with MASLD go on to develop MASH [1]. People with MASH are at a higher risk for developing cirrhosis and ultimately hepatocellular carcinoma (HCC). MASH-associated cirrhosis is the second leading indication and most rapidly increasing indication for liver transplantation in the United States [2,3]. Despite the compelling evidence for immune dysfunction in the development and progression of MASLD to MASH and cirrhosis, the mechanisms driving immune activation and the cellular players contributing to the resulting inflammatory immune response are still unclear.

Recent evidence implicates T cells in promoting hepatic inflammation in MASLD, yet the roles played by various T cell subsets are not well understood [4,5,6]. T cells consist of inflammatory effector and immunosuppressive cell subsets, both with multifaceted roles in tissue injury and repair. In chronic tissue injury, effector T cells produce inflammatory cytokines, chemokines, and cytolytic molecules that perpetuate inflammation and injury. Conversely, immune regulatory T cells suppress these inflammatory responses, promoting tissue repair and limiting tissue injury. The interplay between these T cell subsets ultimately determines the extent of tissue injury in chronic inflammatory diseases, such as MASLD [7,8,9,10].

T regulatory cells (Tregs), a specialized subset of CD4 T cells expressing the transcription factor Foxp3, are crucial for maintaining immune tolerance, preventing autoimmunity, and limiting inflammatory responses in chronic inflammatory diseases. Tregs utilize several mechanisms, such as inhibiting effector T cell proliferation via IL-2 deprivation, secreting inhibitory cytokines like IL-10, IL-35, and TGFβ, and producing adenosine, to suppress T cell activation. Additionally, Tregs use CTLA-4 to block antigen-presenting cell function and granzyme and/or perforin for effector T cell cytolysis. Therefore, loss or functional impairment of Tregs is associated with several immunological and chronic inflammatory diseases [7,11]

Despite their importance in regulating tissue immune homeostasis and suppressing inflammation in chronic inflammatory diseases, the role of Tregs in MASLD progression remains unresolved. Clinical studies in MASLD patients have yielded conflicting results linking both enrichment of and a decrease in hepatic and peripheral Tregs with increased disease severity [12,13,14,15,16,17]. In mouse models in which a high-fat, high-fructose, and high-cholesterol (Western diet, WD) diet was used to induce MASLD, most studies reported enrichment of effector T cells as well as Tregs in the liver [12,18,19,20,21]. Additionally, in the MASH-HCC models, intrahepatic Treg expansion has been shown to promote an immunosuppressive environment conducive to tumor development [22]. However, in mouse models of MASLD and MASH, targeted Treg depletion has produced conflicting results, with some studies reporting exacerbated liver injury and others showing protective effects following depletion [20,23,24]. Additionally, adoptive transfer of CD4+CD25+ T cells exacerbated diet-induced liver injury, while targeted Treg depletion alleviated it, suggesting a potential proinflammatory role of Tregs in the progression of MASLD [20,23]. These contradictory findings underscore the need for definitive studies to understand the role of Tregs in MASLD progression. Here, we utilized orthogonal approaches of conditional T cell depletion, in vivo Treg induction, and ex vivo functional assays to better delineate the function of Tregs in MASLD. Our findings show that the components of WD impair the immunosuppressive function of Tregs, leading to partial suppression of innate and effector T cells and perpetuation of hepatic inflammation in MASLD.

## 2. Methods

### 2.1. Mice

Adult male C57BL/6J and *Rag1^−/−^* mice were obtained from The Jackson Laboratory (Bar Harbor, ME, USA). Foxp3^DTR^ knock-in mice were a gift from Dr. Rafi Ahmed [25]. Age- and sex-matched littermates at six weeks of age were used for all studies. All mice were in C57BL/6 background. Animal studies were performed in accordance with ethical guidelines and regulations set forth by the University of Pittsburgh Institutional Animal Care and Use Committee (IACUC) and conformed to Association for Assessment and Accreditation of Laboratory Animal Care (AAALAC) standards for humane treatment of laboratory animals. All experimental protocols and procedures were reviewed and approved by the IACUC (approval number 21079689, 11 June 2019, PHS Assurance Number: D16-00118). All animal protocols and methods were reported in accordance with ARRIVE (Animal Research: Reporting of In Vivo Experiments) guidelines (https://arriveguidelines.org, (accessed on 20 December 2025)). The mice were housed and cared for at the University of Pittsburgh Division of Animal Resources. The University of Pittsburgh is in compliance with state and federal Animal Welfare Acts.

### 2.2. MASLD Diet

To induce MASLD, six-week-old male C57BL/6J, Foxp3^DTR^, and *Rag1^−/−^* mice and their littermate controls were fed a Western diet (WD) ad libitum for 16 weeks as described previously [4,26]. The WD (TD.130885; Harlan Laboratories) consisted of 0.2% cholesterol, 20% protein, 43% CHO, 23% fat (6.6% trans-fat), and 2.31% fructose [26]. The normal diet (ND) comprised 16% protein, 61% carbohydrate, and 7.2% fat.

### 2.3. Ex Vivo Analysis of Treg Function

Splenic CD4 T cells from WT mice were enriched using a naive untouched CD4 T cell isolation kit as per the manufacturer’s instructions (Miltenyi Biotec, Auburn, CA cat. No. 130-104-453). Isolated CD4 T cells (1 × 10^6^ cells) were incubated at 37 °C with Dynabeads™ Mouse T-Activator CD3/CD28 for T-Cell Expansion and Activation (Thermo scientific, Waltham, MA, USA, cat. No. 11456D) and treated with glucose (25 mM, Fisher Scientific, cat. no. D16-1), fructose (25 mM, Fisher Scientific, Waltham, MA, cat. no. 161355000), or palmitic acid (400 uM, Millipore Sigma, Burlington, MA, cat. no. P0500-10G) for 24 h. Untreated CD4 T cells served as controls. Following 24 h incubation, cells were stimulated with eBioscience Cell Stimulation cocktail (eBioscience, San Diego, CA, USA, cat. No. 00-4970-93) for 1 h, followed by incubation for an additional 4 h in the presence of GolgiPlug (BD Biosciences, cat. No. 555029) and GolgiStop (BD Biosciences, cat. No. 554724) to inhibit cytokine secretion and allow intracellular cytokine accumulation. Cells were stained with a panel of fluorochrome-conjugated antibodies and viability stain (BD Biosciences, San Jose, CA, USA) and acquired on FACS Canto II (BD) equipped with three lasers (see Supplementary Methods). Data were analyzed using FlowJo software (https://www.flowjo.com/) using the gating strategy described in Supplementary Figure S1. Fluorochrome-conjugated antibodies against CD4 (clone RM4-5) and IL10 (clone JES5-16E3) were purchased from BD Biosciences (San Jose, CA, USA), and Foxp3 (clone FJK-16s) and Live/Dead Aqua were purchased from Thermo Fisher Scientific (Rockford, IL, USA).

### 2.4. Treg Depletion

A cohort of Foxp3^DTR^ mice fed a WD for 16 weeks were randomized to receive weekly intraperitoneal injections (IP; 50 μg/kg) of diphtheria toxin (DT) or saline for four weeks starting at week twelve [27]. Diphtheria toxin was purchased from Millipore Sigma (St. Louis, MO, USA) and was reconstituted according to the manufacturer’s protocol (Catalog: D0564).

### 2.5. In Vivo Treg Induction

For the Treg induction studies, we used the established in vivo Treg induction cocktail consisting of recombinant murine IL2 (0.01 ug/gm, Peprotech, Cranbury, NJ, USA, Catalog 212-12) complexed to anti-IL2 antibody (0.05 ug/gm; clone Jes6-1A12; BioXCell, Lebanon, NH, USA) [28]. The cocktail or PBS was administered twice weekly for four weeks, starting at week twelve of WD-feeding.

### 2.6. Human Tissue

Formalin-fixed, de-identified liver tissue sections from explants of disease controls (n = 10) and patients diagnosed with MASH (n = 10) were obtained through the Clinical Biospecimen Repository and Processing Core (CBRPC) at the Pittsburgh Liver Research Center. Control samples consisted of liver tissue taken from parenchyma adjacent to tumors in individuals with hepatocellular carcinoma. The study received approval from the University of Pittsburgh Institutional Review Board (IRB) under the Office of Research Protection, with all procedures following applicable guidelines and regulations (IRB approval number: STUDY20010114, study title: Non-Neoplastic Liver Diseases, approval date 28 February 2020). The requirement for informed consent was waived by the University of Pittsburgh IRB in accordance with the University of Pittsburgh Institutional Review Board guidelines.

### 2.7. Statistical Analysis

One-way ANOVA with post hoc test as well as a two-tailed Student’s *t* test were employed, as appropriate, to investigate statistical differences. A *p* value < 0.05 was considered statistically significant. Data shown are representative of 3 independent experiments. Statistical analyses were performed using Prism 9.0 (San Diego, CA, USA).

## 3. Results

### 3.1. MASLD Progression in Mice Fed a WD Is Associated with Aberrant Activation of Hepatic and Systemic T Cells

**Figure 1:**
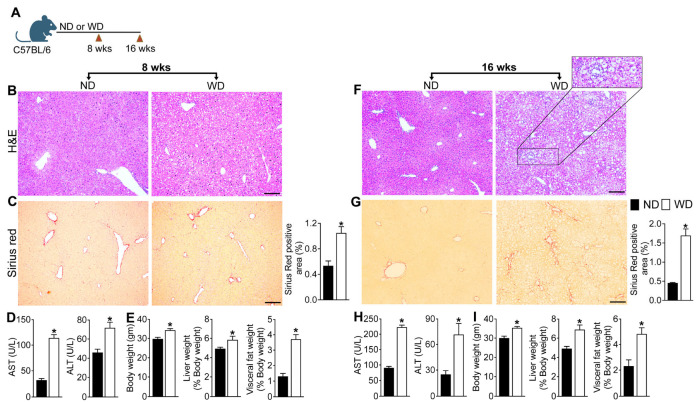
WD-feeding promotes MASLD progression in WT mice. (**A**) Schematic of study design. Six-week-old C57BL/6 J (WT) mice were fed Western diet (WD) or normal diet (ND) for 8 and 16 weeks. Representative photomicrographs of Hematoxylin and Eosin (H&E)- and Sirius red-stained liver tissue sections and quantitative analysis of Sirius red-stained liver tissue sections at (**B**,**C**) eight weeks and (**F**,**G**) sixteen weeks. Scale: 20 um. Serum AST and ALT levels at (**D**) eight weeks and (**H**) sixteen weeks. Body weight and liver and visceral fat weight expressed as percentage of body weight at (**E**) eight weeks and (**I**) sixteen weeks. Data are representative of 3 independent experiments (n = 5–8 mice per group). In the bar graphs, black bars represent ND-fed animals and white bars represent WD-fed animals; data are shown as mean ± SEM. Asterisks indicate significant differences (*p* < 0.05) between ND- and WD-fed mice.

To establish a diet-induced model of MASLD progression, we examined liver histology as well as biochemical markers of MASLD in C57B/6J mice fed a WD for eight weeks (early disease) or sixteen weeks (advanced disease) (Figure 1A–I). Eight weeks of WD consumption significantly increased body weight as well as liver and visceral fat weight expressed as percentage of body weight (Figure 1E). Histologic staining of the liver tissue revealed mild centrilobular micro- and macro-vesicular steatosis as well as mild lobular inflammation indicated by lobular infiltration of immune cells (Figure 1B). Serum AST and ALT levels were significantly higher in the mice fed a WD for eight weeks relative to ND-fed mice (Figure 1D). However, eight weeks of WD-feeding did not induce hepatic fibrosis as indicated by the absence of a marked increase in collagen deposition in the liver tissue sections stained with Sirius red (Figure 1C). In contrast, mice fed a WD for sixteen weeks developed severe hepatosteatosis and prominent hepatocyte ballooning and lobular inflammation, demonstrating the progressive nature of the disease (Figure 1F). Sixteen weeks of WD-feeding enhanced hepatic fibrosis, indicated by increased collagen deposition in the Sirius red-stained liver tissue sections (Figure 1G). Consistent with the liver pathology, sixteen weeks of WD-feeding further increased serum ALT and AST levels, suggesting significant disease progression (Figure 1H). Disease progression in WD-fed mice correlated with increased body weight as well as liver and fat weight expressed as percentage of body weight (Figure 1I). Together these data demonstrate that WD-fed WT mice serve as a progressive model of MASLD and develop key histologic and biochemical features of human MASH including hepatosteatosis, inflammation, and fibrosis when fed a WD for sixteen weeks. It should be noted that MASLD is a complex heterogeneous disease and MASLD progression in human adults requires prolonged consumption of an unhealthy diet. Thus, while WD-fed WT mice may not represent the entire spectrum of human MASLD, the model closely recapitulates histopathologic, biochemical, and metabolic characteristics of MASLD progression in human adults.

**Figure 2:**
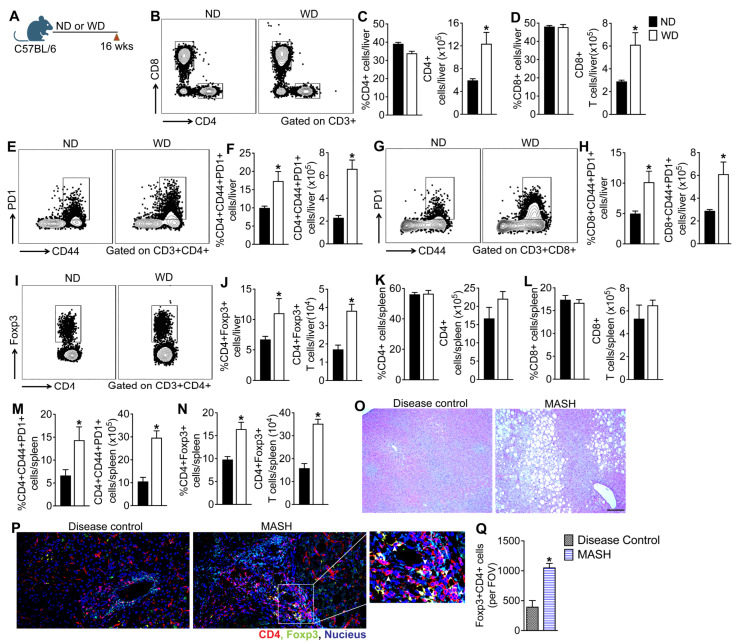
MASLD progression in mice fed a Western diet is associated with aberrant activation of hepatic and systemic T cells. (**A**) Schematic of study design. WT mice were fed an ND or WD for sixteen weeks and intrahepatic lymphocytes were analyzed by FACS. (**B**) Representative flow plots show the percent of CD4 T cells in the liver. Bar graphs show the percent and total number of (**C**) CD4 and (**D**) CD8 T cells in the liver (n = 5 mice per group). (**E**) Representative flow plots show the percent of PD-1+CD44+ CD4 T cells and (**F**) bar graphs show the percent and total number of PD-1+CD44+ CD4 T cells in the liver. (**G**) Representative flow plots show the percent of PD-1+CD44+ CD8 T cells and (**H**) bar graphs show the percent and total number of PD-1+CD44+ CD8 T cells in the liver. (**I**) Representative flow plots show the percent of Foxp3+ CD4 T cells and (**J**) bar graphs show the percent and total number of Foxp3+ CD4 T cells in the liver. Bar graphs show the percent and total number of (**K**) CD4 and (**L**) CD8 T cells in the spleen. Bar graphs show the percent and total number of (**M**) PD-1+CD44+ CD4 and (**N**) PD-1+CD44+ CD8 T cells in the spleen. Data are representative of 3 independent experiments (n = 5–8 mice per group). In the bar graphs, black bars represent ND-fed animals and white bars represent WD-fed animals; data are shown as mean ± SEM. (**O**) Representative photomicrographs of Hematoxylin and Eosin (H&E), (**P**) representative confocal images of Foxp3+ cells (green) and CD4 (red) in the liver tissue sections from disease controls and MASH patients, and (**Q**) quantification of Foxp3+CD4+ cells per field of view (FOV) from the confocal images (n = 10 subjects per group). Red arrowheads indicate CD4+FoxP3+ cells and white arrowheads indicate CD4+ T cells. Nuclei are stained blue. Scale: 20 um. Asterisks indicate significant differences (*p* < 0.05) between ND- and WD-fed mice.

After establishing that WD-fed WT mice serve as a progressive model of MASLD that closely recapitulates human disease, we next examined intrahepatic lymphocytes in mice fed the WD for sixteen weeks (Figure 2A, Supplementary Figure S1A). Given prior preclinical studies, including our own, implicating T cells in MASLD progression, we focused our analysis on the T cell compartment [4,12,18,19,20]. In contrast to previous reports, refs. [23,29] WD-feeding did not alter the percentage of intrahepatic CD4 and CD8 T cells but significantly increased the total numbers of CD4 and CD8 T cells in the liver of WD-fed mice relative to ND-fed controls (Figure 2B–D). Further analysis revealed the enrichment of CD4 T cells expressing T cell activation markers CD44 and PD-1 in the livers of WD-fed mice, suggesting that WD consumption increases hepatic CD4 T cell activation (Figure 2E–H). Interestingly, WD-feeding also increased the percentage and total number of Foxp3+ T regulatory (Tregs) cells in the liver (Figure 2I,J). In contrast to the liver, we did not observe changes in either percentages or total numbers of CD4 and CD8 T cells in the spleen, suggestive of enhanced T cell recruitment to the liver (Figure 2K,L). WD-feeding did, however, enrich CD44 and PD-1 double positive CD4 T cells in the spleen of WD-fed mice, which paralleled the enrichment of Foxp3+ Treg cells in the spleen (Figure 2M,N). To determine whether Foxp3+ CD4 T cells are enriched in the livers of patients with metabolic dysfunction-associated steatohepatitis (MASH), we analyzed formalin-fixed, de-identified liver tissue sections from disease controls (n = 10) and patients with MASH (n = 10). Sections were co-stained for CD4 and Foxp3 and analyzed by confocal microscopy. In agreement with previous reports, we also observed enrichment of CD4+Foxp3+ Treg cells in liver tissue sections from MASH patients compared to disease controls, which suggests that Foxp3+ Treg cell enrichment also occurs in human MASH (Figure 2O–Q) [14]. Interestingly, most of the CD4+Foxp3+ Tregs are localized to fibrotic portal areas and are in close proximity to CD4+ T cells. Collectively, these data suggest that WD-feeding results in the heightened activation of T cells, which parallels a compensatory increase in Foxp3+ Treg cells in the liver.

### 3.2. Myeloid Cells Promote Hepatic Inflammation and Fibrosis in the Absence of Lymphoid Cells

**Figure 3:**
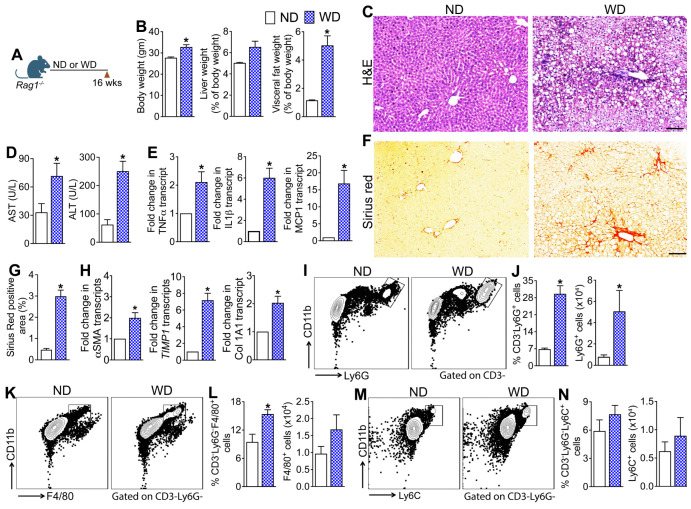
Myeloid cells promote hepatic inflammation and fibrosis in the absence of lymphoid cells. (**A**) Schematic of study design. Rag1 KO mice were fed an ND or WD for sixteen weeks. (**B**) Body weight and liver and visceral fat weight expressed as a percentage of body weight following sixteen weeks of WD-feeding. (**C**) Representative photomicrographs of Hematoxylin and Eosin (H&E)-stained liver tissue sections. (**D**) Serum AST and ALT levels. (**E**) Transcript levels of key inflammatory cytokines in the liver. (**F**) Representative photomicrographs of Sirius red-stained liver tissue sections. (**G**) Quantitative analysis of Sirius red-stained liver tissue sections. (**H**) Transcript levels of key markers of liver fibrosis. (**I**) Representative flow plots show the percent of neutrophils (CD11b+Ly6G+ cells) and (**J**) bar graphs show the percent and total number of neutrophils in the liver. (**K**) Representative flow plots show the percent of macrophages (CD11b+F4/80+ cells) and (**L**) bar graphs show the percent and total number of macrophages in the liver. (**M**) Representative flow plots show the percent of monocytes (CD11b+Ly6C+ cells) and (**N**) bar graphs show the percent and total number of monocytes in the liver. Data are representative of 2 independent experiments (n = 5–8 mice per group). In the bar graphs, white bars represent ND-fed animals and blue bars represent WD-fed animals; data are shown as mean ± SEM. Scale: 20 um. Asterisks indicate significant differences (*p* < 0.05) between ND- and WD-fed mice.

To examine the contribution of adaptive immune cells in promoting MASLD progression, we fed the WD to *Rag1^−/−^* mice that lack functional T and B cells (Figure 3A). Following sixteen weeks of WD-feeding, *Rag1^−/−^* mice gained significant body weight, and both liver weight/body weight and visceral fat weight/body weight ratios were significantly higher relative to ND-fed mice (Figure 3B). Histologic images of the liver tissue sections stained with H&E revealed micro- and macro-vesicular steatosis, portal inflammatory infiltrates, and ballooning degeneration of hepatocytes, mirroring the WT mice fed the WD for sixteen weeks (Figure 3C and Figure 1F). Liver injury in WD-fed *Rag1^−/−^* mice was further validated by higher serum AST and ALT levels relative to the ND-fed mice, suggesting that WD-feeding induced significant hepatic injury in the *Rag1^−/−^* mice (Figure 3D). Transcript levels of key inflammatory cytokines, TNFα and IL-1β, and chemokine, MCP1 were also significantly higher in the WD-fed *Rag1^−/−^* mice relative to ND-fed controls (Figure 3E). As shown in Figure 3F,G, WD-fed *Rag1^−/−^* mice also developed extensive pericentral, periportal, and sinusoidal fibrosis. Increased hepatic fibrosis in WD-fed *Rag1^−/−^* mice was corroborated by a significant increase in the transcript levels of key markers of hepatic fibrosis, including αSMA, TIMP1, and Col1A1 (Figure 3H).

To identify the immune cells contributing to hepatic inflammation and fibrosis in the absence of adaptive immune cells, we analyzed hepatic immune infiltrates in the WD-fed *Rag1^−/−^* mice (Figure 3I–N). Our phenotypic analyses revealed significant enrichment of neutrophils (CD3-Ly6G+ cells) (Figure 3I,J) and macrophages (CD3-Ly6G-F4/80+ cells) (Figure 3K,L) but not monocytes (CD3-Ly6G-Ly6C+ cells) (Figure 3M,N) in the liver of WD-fed *Rag1^−/−^* mice relative to ND-fed controls. Collectively, these data suggest that in the absence of adaptive immune cells, myeloid cells promote WD-induced hepatic inflammation and fibrosis, resulting in the development and progression of MASLD.

### 3.3. Depletion of Foxp3+ Regulatory T Cells Reduces Hepatic Steatosis but Exacerbates WD-Induced Hepatic Inflammation

**Figure 4:**
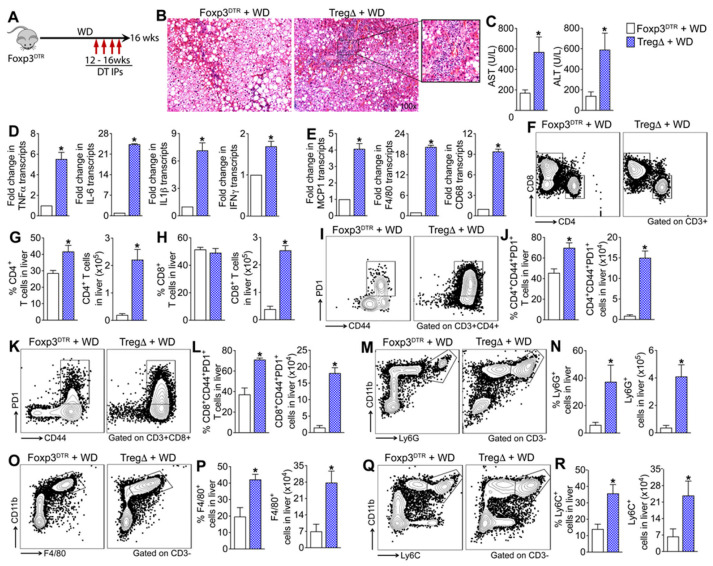
Depletion of Foxp3+ regulatory T cells reduces hepatic steatosis but exacerbates Western diet-induced hepatic inflammation. (**A**) Schematic of study design. A cohort of Foxp3^DTR^ mice fed a WD for sixteen weeks were randomized to receive weekly intraperitoneal injections of diphtheria toxin (DT, TregΔ) or saline (controls) for four weeks starting at week twelve. (**B**) Representative photomicrographs of Hematoxylin and Eosin (H&E)-stained liver tissue sections. (**C**) Serum AST and ALT levels. (**D**,**E**) Transcript levels of key inflammatory cytokines, chemokines, and markers of monocytes and macrophages in the liver. (**F**) Representative flow plots show the percent of intrahepatic CD4 and CD8 T cells. Bar graphs show the percent and total number of intrahepatic (**G**) CD4 and (**H**) CD8 T cells. (**I**) Representative flow plots show the percent of intrahepatic PD-1+CD44+ CD4 T cells and (**J**) bar graphs show the percent and total number of PD-1+CD44+ CD4 T cells. (**K**) Representative flow plots show the percent of intrahepatic PD-1+CD44+ CD8 T cells and (**L**) bar graphs show the percent and total number of intrahepatic PD-1+CD44+ CD8 T cells. (**M**) Representative flow plots show the percent of intrahepatic neutrophils (CD11b+Ly6G+ cells) and (**N**) bar graphs show the percent and total number of intrahepatic neutrophils. (**O**) Representative flow plots show the percent of intrahepatic macrophages (CD11b+F4/80+ cells) and (**P**) bar graphs show the percent and total number of intrahepatic macrophages. (**Q**) Representative flow plots show the percent of intrahepatic monocytes (CD11b+Ly6C+ cells) and (**R**) bar graphs show the percent and total number of intrahepatic monocytes. Data are representative of 3 independent experiments (n = 5 mice per group). In the bar graphs, white bars represent WD-fed Foxp3^DTR^ mice and blue bars represent WD-fed TregΔ mice; data are shown as mean ± SEM. Scale: 20 um. Asterisks indicate significant differences (*p* < 0.05) between TregΔ and control mice.

Having established that the absence of adaptive immune cells does not offer protection against WD-induced liver injury and that WD consumption results in enrichment of systemic and hepatic Treg cells, we next investigated whether Foxp3+ Treg cells play a role in MASLD progression by suppressing aberrant activation of innate and adaptive immune cells. To specifically investigate the role of Treg cells in MASLD progression, we used *Foxp3*^DTR^ knock-in mice in which Foxp3^+^ Treg cells express human diphtheria toxin receptor (DTR) and green fluorescence protein (GFP) under the control of Foxp3 transcriptional regulatory element (Foxp3^DTR^) that allows specific depletion of Treg cells in vivo. Insertion of the IRES-DTR-GFP cassette in the *Foxp3* locus does not affect Foxp3 gene expression or Treg cell function [25]. Additionally, DT-induced cell death is apoptotic and therefore does not initiate a proinflammatory immune response [30,31,32], rendering this a robust model to study the effects of WD-induced inflammation in MASLD pathogenesis. For this study, a cohort of Foxp3^DTR^ mice and DTR-negative (WT) mice fed a WD for sixteen weeks were randomized to receive weekly intraperitoneal injections (50 μg/kg) of DT or saline for four weeks starting at week twelve [27] (Figure 4A, Supplementary Figure S3A). We used this standardized regimen as it is effective in depleting Tregs without inducing significant pathology in non-DTR mice [27]. As shown in Supplementary Figure S2A,B, four weeks of DT treatment was effective in significantly reducing intrahepatic Tregs in the WD-fed Foxp3^DTR^ mice. Histologic analysis of liver tissue sections revealed milder steatosis in Treg-depleted (TregΔ) mice fed a WD relative to control mice but more severe hepatic inflammation as indicated by increased infiltration of immune cells (Figure 4B). Serum AST and ALT levels, as well as the transcript levels of key proinflammatory cytokines and chemokines, TNFα, IL6, IL-1β, IFNγ, and MCP1, and markers of monocytes and macrophages, CD68 and F4/80, were significantly higher in the WD-fed TregΔ mice relative to controls. No differences in liver injury were observed between WD-fed WT mice treated with DT or saline, indicating that DT administration in WT mice does not contribute to hepatic injury (Supplementary Figure S3B–D). Together these data suggest that depletion of Treg cells exacerbated WD-induced hepatic inflammation and injury (Figure 4C–E).

Phenotypic analyses of the hepatic immune infiltrates revealed significant enrichment of intrahepatic CD4 and CD8 T cells in the WD-fed TregΔ mice relative to controls (Figure 4F–H). Further analysis revealed significant enrichment of CD44 and PD-1 double positive activated CD4 and CD8 T cells in the liver (Figure 4I–L). Analysis of the intrahepatic innate immune cells revealed significant enrichment of neutrophils (CD3-Ly6G+ cells), monocytes (CD3-Ly6G-Ly6C+ cells), and macrophages (CD3-Ly6G-F4/80+ cells) in the WD-fed TregΔ mice relative to controls (Figure 4M–R). No differences in intrahepatic innate and adaptive immune cell populations or in their activation status were observed between WD-fed WT mice treated with DT or saline, indicating that DT administration in WT mice does not contribute to hepatic inflammation (Supplementary Figure S3E–Q). Collectively, these data demonstrate that Tregs suppress aberrant activation and infiltration of adaptive and innate immune cells in the liver of WD-fed mice and protect the liver from WD-induced inflammation and injury.

### 3.4. Depletion of Foxp3+ Regulatory T Cells Exacerbates WD-Induced Hepatic Fibrosis

**Figure 5:**
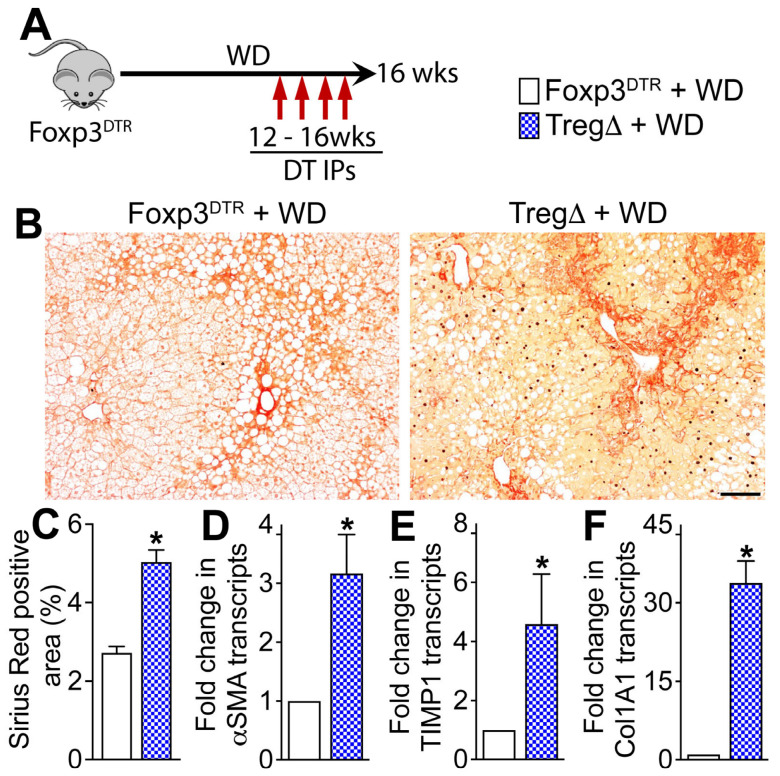
Depletion of Foxp3+ regulatory T cells exacerbates WD-induced hepatic fibrosis. (**A**) Schematic of study design. A cohort of Foxp3^DTR^ mice fed a WD for sixteen weeks were randomized to receive weekly intraperitoneal injections of diphtheria toxin (DT, TregΔ) or saline (controls) for four weeks starting at week twelve. (**B**) Representative photomicrographs of Sirius red-stained liver tissue sections. (**C**) Quantitative analysis of Sirius red-stained liver tissue sections. (**D**–**F**) Transcript levels of key liver fibrosis markers in the liver. Data are representative of 3 independent experiments (n = 5 mice per group). In the bar graphs, white bars represent WD-fed Foxp3^DTR^ mice and blue bars represent WD-fed TregΔ mice; data are shown as mean ± SEM. Scale: 20 um. Asterisks indicate significant differences (*p* < 0.05) between TregΔ and control mice.

To determine the effect of Treg depletion on WD-induced hepatic fibrosis, we assessed liver tissue sections for the presence of collagen and quantified transcript levels of key hepatic fibrosis markers (Figure 5A–F). As seen in Figure 5B,C, WD consumption resulted in more severe hepatic fibrosis in TregΔ mice relative to controls. Consistent with higher hepatic fibrosis in TregΔ mice relative to controls, transcript levels of key liver fibrosis markers, αSMA, TIMP-1, and Col1A1, were also significantly higher in the TregΔ mice (Figure 5D–F). Together, these data demonstrate that Foxp3+ Tregs play a role in the mitigation of hepatic inflammation as well as fibrosis in WD-fed mice.

### 3.5. Induction of Tregs in WD-Fed Mice Attenuates Hepatic Inflammation and Steatosis

**Figure 6:**
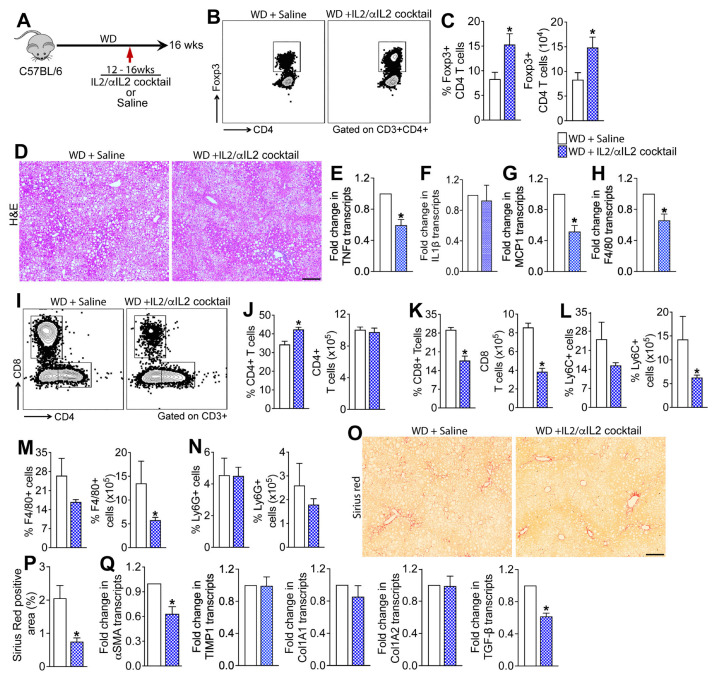
Induction of Tregs in Western diet-fed mice attenuates hepatic inflammation and fibrosis. (**A**) Schematic of study design. A cohort of WT mice fed a WD for sixteen weeks were randomized to receive twice weekly injections of Treg induction cocktail, consisting of recombinant murine IL2 complexed to anti-IL2 antibody, or saline for four weeks starting at week twelve. (**B**) Representative flow plots show the percent of intrahepatic Foxp3+ CD4 T cells and (**C**) bar graphs show the percent and total number of intrahepatic Foxp3+ CD4 T cells. (**D**) Representative photomicrographs of Hematoxylin and Eosin (H&E)-stained liver tissue sections. (**E**–**H**) Transcript levels of key inflammatory cytokines, chemokines, and markers of macrophages in the liver. (**I**) Representative flow plots show the percent of intrahepatic CD4 and CD8 T cells. Bar graphs show the percent and total number of intrahepatic (**J**) CD4 and (**K**) CD8 T cells. Bar graphs show the percent and total number of intrahepatic (**L**) neutrophils (CD11b+Ly6G+ cells), (**M**) monocytes (CD11b+Ly6C+ cells), and (**N**) macrophages (CD11b+F4/80+ cells). (**O**) Representative photomicrographs of Sirius red-stained liver tissue sections. (**P**) Quantitative analysis of Sirius red-stained liver tissue sections. (**Q**) Transcript levels of key liver fibrosis markers in the liver. Data are representative of 2 independent experiments (n = 5 mice per group). In the bar graphs, white bars represent saline-treated WD-fed mice and blue bars represent IL2/αIL2 cocktail-treated WD-fed mice; data are shown as mean ± SEM. Scale: 20 um. Asterisks indicate significant differences (*p* < 0.05) between saline- and IL2/αIL2 cocktail-treated mice.

Having established that Tregs suppress WD-induced hepatic inflammation and fibrosis, we next investigated whether Treg expansion would prevent MASLD progression in WT mice fed a WD for sixteen weeks. We used the established in vivo Treg expansion cocktail, recombinant murine IL2 complexed to anti-IL2 mAb (IL2/αIL2 cocktail), to expand Treg cells in mice fed a WD for sixteen weeks (Figure 6A). As shown in Figure 6B,C, four weeks of treatment with IL2/αIL2 cocktail resulted in ~2-fold expansion of intrahepatic Foxp3+ Tregs in the WD-fed WT mice. Assessment of liver histopathology in H&E-stained liver sections revealed marked reduction in steatosis as well as hepatic inflammation in WD-fed WT mice treated with IL2/αIL2 cocktail relative to saline-treated animals (Figure 6D). Reduced hepatic inflammation in the IL2/αIL2 cocktail-treated mice was further validated by a significant reduction in the transcript levels of TNFα, MCP-1, and F4/80 in the liver, but no changes in IL-1β were observed (Figure 6E–H). Analysis of the intrahepatic immune cells revealed that the treatment with IL2/αIL2 cocktail significantly increased the percentage of CD4 T cells but did not alter the total number of CD4 T cells in the liver (Figure 6I,J). Interestingly, IL2/αIL2 cocktail treatment significantly reduced both the percentage and total number of intrahepatic CD8 T cells (Figure 6K). Treatment with IL2/αIL2 cocktail also reduced the percentages and total numbers of Ly6C+ monocytes and F4/80+ macrophages; however, the reduction in the percentages of monocytes and macrophages did not reach statistical significance (Figure 6L–M). No changes in the percentage and total number of Ly6G+ neutrophils were observed between IL2/αIL2 cocktail- vs. saline-treated mice (Figure 6N). Collectively these data suggest that augmenting the hepatic Treg population affords protection from WD-induced inflammation and steatosis by reducing the populations of adaptive and innate immune cells in the liver.

### 3.6. Treg Expansion Therapy Protects Mice from WD-Induced Liver Fibrosis

Assessment of collagen deposition in Sirius red-stained liver sections revealed marked reduction in collagen deposition in the WD-fed WT mice treated with IL2/αIL2 cocktail relative to saline-treated animals (Figure 6O,P). Consistent with lower hepatic fibrosis in IL2/αIL2 cocktail-treated animals relative to controls, transcript levels of key liver fibrosis markers, αSMA and TGF-β, were significantly lower in the liver, but no differences in TIMP-1, Col1A1, and Col1A2 were observed (Figure 6Q). Together, these data suggest that augmenting the hepatic Treg population attenuates WD-induced hepatic fibrosis by suppressing HSC activation.

### 3.7. Dietary Glucose and Palmitate Impair Hepatic Treg Function

**Figure 7:**
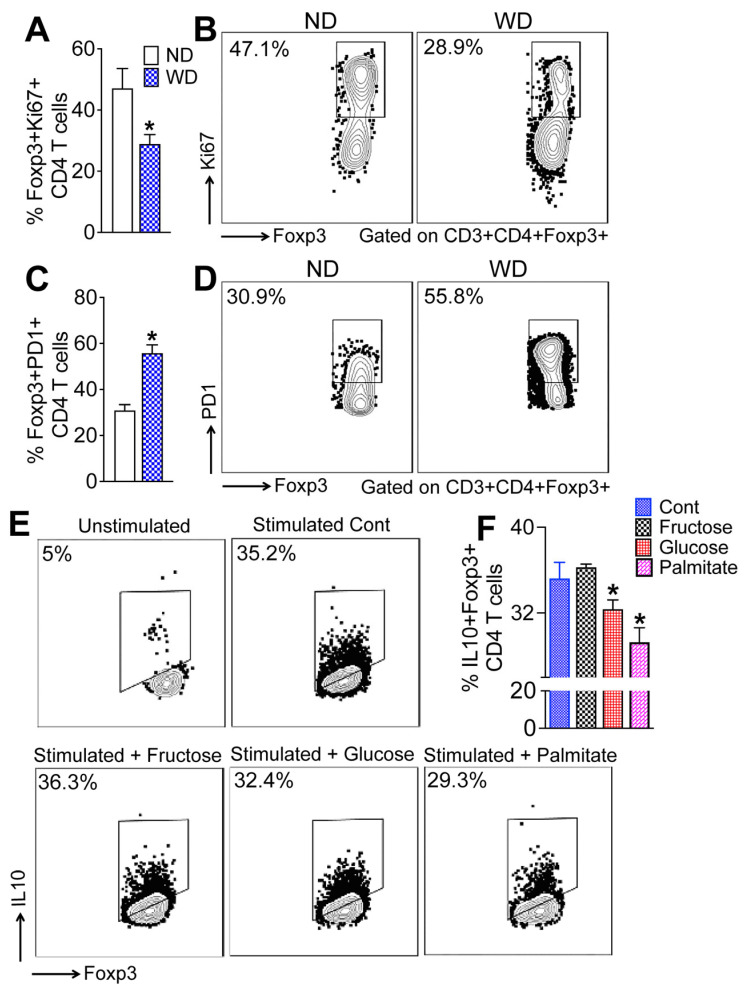
Dietary glucose and palmitate impair hepatic Treg function. (**A**) Bar graphs and (**B**) representative flow plots show the percent of FoxP3+ Ki67+ CD4 T cells in the liver of WT mice fed an ND or WD for sixteen weeks. (**C**) Bar graphs and (**D**) representative flow plots show the percent of FoxP3+ PD-1+ CD4 T cells in the liver of WT mice fed an ND or WD for sixteen weeks. Data are representative of 2 independent experiments (n = 5 mice per group). Data are presented as mean ± SEM. Asterisks indicate significant differences (*p* < 0.05) between ND- and WD-fed mice. (**E**) Representative flow plots show the percent of FoxP3+ IL10+ CD4 T cells and (**F**) bar graph shows the percent of FoxP3+ IL10+ CD4 T cells following treatment with fructose, glucose, and palmitate. Splenic CD4 T cells were treated with glucose, fructose, or palmitic acid for 24 h and IL10 production was detected by FACS. Data are representative of 3 independent experiments (n = 3 reps. per treatment). Data are presented as mean ± SEM. Asterisks indicate significant differences (*p* < 0.05) between stimulated controls and treated cells.

To assess the functional state of hepatic Tregs during MASLD progression, we analyzed proliferation and inhibitory receptor expression in intrahepatic Foxp3+ Treg cells from WD-fed mice. As shown in Figure 7A,B, a significantly lower percentage of Foxp3+ Treg cells in the livers of WD-fed mice, relative to the ND-fed controls, expressed the proliferation marker Ki67, indicating reduced Treg proliferation. Since PD-1-expressing Treg cells are less proliferative and immunosuppressive [33], we examined PD-1 expression in the hepatic Treg cells following WD-feeding. We found that a higher percentage of Foxp3+ Treg cells in the liver of WD-fed mice expressed PD-1 relative to the ND-fed mice (Figure 7C,D). These data suggest that WD-feeding impairs hepatic Treg function and provide an explanation for the inability of Tregs to suppress WD-induced hepatic inflammation.

Since a tissue microenvironment that enhances glycolysis and anabolic metabolism in Treg cells impairs the proliferative and immunosuppressive ability of Tregs [34,35], we performed ex vivo experiments with mouse primary splenic lymphocytes to determine whether excess glucose, fructose, or palmitic acid can impair Treg function (Figure 7E,F). CD4 T cells were isolated from WT mice, activated ex vivo, and exposed to elevated concentrations of glucose, fructose, or palmitic acid, followed by intracellular cytokine staining to assess IL-10 production by Foxp3+ Treg cells as a functional readout of immunosuppressive capacity. We found that the percentage of IL10+ Treg cells was significantly reduced when splenic lymphocytes were cultured in high concentration of glucose or palmitic acid (Figure 7E,F). Lymphocytes cultured in high concentrations of fructose did not affect the percentage of IL10+ Tregs (Figure 7E,F). Together, these data suggest that dietary glucose and palmitic acid impair IL10 production by hepatic Tregs, resulting in the inability of Tregs to suppress WD-induced hepatic inflammation in MASLD.

## 4. Discussion

In this study, we utilized orthogonal approaches comprising conditional T cell depletion, targeted Treg reduction, and in vivo Treg induction to elucidate the role Tregs play in MASLD progression. Our data support three major conclusions: First, Tregs actively infiltrate the liver during MASLD progression and suppress activation of innate and adaptive hepatic immune cells, thereby resolving inflammation. Second, the immunosuppressive role of Tregs attenuates liver injury, preventing rapid progression of MASLD. Thirdly, we report functional impairment of Tregs in MASLD, a process driven by dietary glucose and fatty acids. Our data suggest that Treg dysfunction results in unfettered immune activation, perpetuating chronic inflammation that drives disease progression. Altogether, these findings address an unresolved and important question in MASLD research concerning the role of Tregs in preventing disease progression.

By leveraging a diet-induced model of progressive MASLD, we observed a significant increase in T cell activation in the spleen suggestive of WD-induced systemic T cell activation. These data are consistent with prior studies, including our own, demonstrating that increased intestinal epithelial permeability in MASLD drives gut microbial translocation and promotes systemic inflammation [26]. In the present study, we observed increased accumulation of effector and regulatory CD4 T cells in the liver, suggesting enhanced T cell recruitment in driving hepatic inflammation and fibrosis in MASLD. These findings are consistent with our studies, demonstrating a role for integrin α_4_β_7_ and its ligand mucosal addressin cell adhesion molecule (MAdCAM)-1 in effector T cell recruitment to the MASLD liver [4]. WD consumption also induces a systemic increase in Tregs, with a larger population of Tregs subsequently accumulating in the liver [4]. Collectively these results, along with recent published reports from other groups, as well as our own, support a direct role for effector T cells and Tregs in MASLD progression [4,17,19].

Our first approach to parse out the role of adaptive immune cells in MASLD was to use *Rag-1* KO mice that lack functional T and B cells. Reports of both protection and exacerbation of hepatic injury in Rag-1 KO mice led us to reexamine MASLD development in this model and understand the effect of diet on the myeloid compartment in the absence of functional adaptive immune cells [20,36,37]. We found that Rag-1 KO mice fed a WD for sixteen weeks develop more severe hepatic inflammation and fibrosis compared to WT mice. As expected, hepatic injury in WD-fed Rag-1 KO mice was driven by myeloid cells, in particular neutrophils and macrophages. These data imply that the adaptive immune cells offer hepatoprotection from diet-induced liver injury by suppressing exorbitant activation of myeloid cells. Similar to our findings, a recent study reported exacerbation of hepatic injury in WD-fed BALB/c Rag-1 KO mice [20]. In contrast, previous studies reported protection from a choline-deficient high-fat diet (CD-HFD) or fructose-induced liver injury in *Rag-1* KO mice [36,37]. These disparate results may likely be explained by the differences in the mechanisms of liver injury induced by diet composition or the genetic background of the mouse model; however, further research is needed to delineate underlying mechanisms.

Our findings that targeted Treg depletion exacerbates WD-induced hepatic inflammation and fibrosis, thereby accelerating MASLD progression, align with studies showing a similar exacerbation of MCD-induced liver injury in Treg-depleted mice [24]. This supports an immunosuppressive role for Tregs in MASLD. In contrast, a recent study reported protection from CD-HFD-induced hepatic injury in targeted Treg-depleted mice, suggesting a permissive rather than protective role for Tregs in a CD-HFD-fed model of MASH [22]. The same study used evidence that the anti-CD25 antibody protects mice from CD-HFD-induced hepatic injury to suggest a proinflammatory role of Tregs in MASH pathogenesis [22]. It is difficult to conclude the proinflammatory role of Tregs in MASH based on these data alone, as CD25 is highly expressed by effector T cells and anti-CD25 antibodies deplete these CD25-expressing effector T cells [38]. Similarly, adoptive transfer of CD4+CD25+ T cells to study Treg function is not a specific approach to delineate Tregs, as CD25+ CD4 T cells can differentiate into both effector T cells and Tregs, a well-documented threat to Treg adoptive transfusion therapies [39,40]. The fate of adoptively transferred Tregs in the tissue is influenced by tissue microenvironment, dietary nutrients, vitamins, and metabolites from both the host and microbiota [39,41,42,43,44]. Moreover, due to the plasticity of Foxp3^+^ Tregs in inflammatory conditions and their functional complexity in both suppressing inflammation and promoting tissue repair, Treg depletion or adoptive transfer may not faithfully recapitulate their multifaceted role in chronic liver diseases [17,45]. While thymic-derived Tregs with a fully demethylated Foxp3 Treg-specific demethylated region (TSDR) are relatively stable, their phenotype and function are strongly influenced by local cytokine and metabolic cues in inflammatory environments. Proinflammatory cytokines such as IFNγ, IL-6, and IL-1, which are elevated in inflammatory settings including MASH, induce T-bet and RORγt while repressing Foxp3, thereby promoting the emergence of IFNγ- or IL-17A- producing proinflammatory “exTregs”. Thus, under inflammatory conditions such as MASLD/MASH, it becomes difficult to determine whether adoptively transferred Tregs maintain a regulatory phenotype in vivo or contribute to ongoing inflammation through conversion to effector T cells [41,42,43,44]. Additionally, Tregs exhibit a dual role in fibrosis, acting as both suppressors and promoters of fibrogenesis depending on the stage of tissue damage, further complicating interpretation of such experimental approaches [17,45].

Our in vivo Treg augmentation studies offer clearer evidence of the beneficial role of Tregs in MASLD progression, showing that expanding the Treg population reduces hepatic inflammation and fibrosis. This finding is consistent with observations in sclerosing cholangitis (SC), where Treg augmentation attenuates hepatic inflammation and fibrosis [46]. It is likely that Tregs play an anti-inflammatory role in the early stages of MASLD, producing cytokines like IL-10 and TGF-β, which suppress immune responses and mitigate liver injury [17,45]. However, as MASLD progresses, Tregs may adopt a profibrogenic role, particularly through the production of TGF-β and amphiregulin, which activate hepatic stellate cells and promote fibrosis [17,20,21,22,23,45]. Therefore, further in-depth studies are required to clarify the specific contributions of Tregs at different stages of MASLD progression, especially their potential transition from a protective to a profibrogenic role as the disease advances.

To understand whether dietary factors might drive Treg dysfunction in MASLD, we focused our attention on ex vivo studies. Cell-extrinsic factors, including nutrients, vitamins, and metabolites, as well as cell-intrinsic metabolic programs, play a critical role in regulating functional stability, plasticity, and tissue-specific heterogeneity of Tregs. Unlike Th1, Th2, and Th17 cells, Treg cells are less reliant on glycolysis and use mitochondrial metabolism and oxidative phosphorylation (OXPHOS) for energy production [47]. In fact, induction of glycolysis in Tregs has been shown to negatively affect immunosuppressive function of Tregs [34,48,49]. In line with the role of metabolism in modulating Treg function, our in vivo studies revealed that Tregs in the MASLD liver express PD-1 and are less proliferative, suggesting a suppressive liver microenvironment. In line with our in vivo data, our ex vivo studies revealed that spiking culture media with glucose and palmitic acid, but not fructose, impairs the ability of Tregs to produce IL10, suggesting that glucose impairs immunosuppressive function of Tregs. While the role of fructose in Treg function should be further explored, no observed effect of fructose on Treg function can be due to the differences in the pathways involved in the cellular metabolism of glucose and fructose. Supporting this, a recent study showed that monocytes treated with high levels of fructose have reduced glycolytic rate and enhanced OXPHOSs [50], metabolic pathways favored by Tregs for energy production [51]. Our findings showing functional impairment of Tregs by palmitic acid is surprising given the ability of Tregs to utilize lipids for energy and maintain functional stability in tumor microenvironments containing high levels of fatty acids [52]. However, studies on similar tumor models showed a lack of increased fatty acid uptake by tumor-infiltrating Tregs and the ability of Tregs to utilize both glycolytic and oxidative metabolism for survival in the tumor [52]. This demonstrates the inherent plasticity of Tregs to acquire tissue-specific metabolic function and the complexity and heterogeneity of metabolic pathways in modulating Treg proliferation and function. Thus, further studies are needed to explore the metabolic regulation of Tregs in MASLD.

In summary, our study provides evidence supporting an anti-inflammatory role of Tregs in MASLD pathogenesis. Our in vivo and ex vivo studies revealed that glucose and palmitic acid impair the immunosuppressive function of Tregs in MASLD, leading to partial suppression of effector immune cells and perpetuation of hepatic inflammation. However, compensatory recruitment and accumulation of Tregs in the liver prevent rapid progression of MASLD despite their functional impairment. Collectively, our work uncovers a novel mechanism by which dietary components prevent immune regulation by Tregs, allowing chronic inflammation to persist in MASLD.

### Limitations of the Study

Although the WD-fed mouse models used here recapitulate core metabolic, histologic, biochemical, and immunologic features of MASLD, they do not capture the full heterogeneity of human disease. Additionally, our analyses provide a cross-sectional assessment of Treg function in early MASLD, limiting insight into how Treg function evolves with disease progression to fibrosis, cirrhosis, and HCC. While ex vivo experiments demonstrate that excess glucose and fatty acids impair Treg function, these assays do not recapitulate the full complexity of the hepatic microenvironment. Future longitudinal studies are needed to determine how metabolic and inflammatory cues regulate Treg stability and function at different stages of MASLD progression and whether these changes persist in advanced disease.

## Data Availability

The original contributions presented in this study are included in the article/Supplementary Materials. Further inquiries can be directed to the corresponding author.

## Supplementary Materials

The following supporting information can be downloaded at https://www.mdpi.com/article/10.3390/cells15020165/s1, Figure S1: Gating strategy used to identify intrahepatic lymphoid and myeloid cell subsets; Figure S2: Depletion of Foxp3+ regulatory T cells in Foxp3DTR mice; Figure S3: No differences in hepatic inflammation and fibrosis in WD-fed DTR-negative mice treated with DT.
