# Supplementary material for: Western Diet Dampens T Regulatory Cell Function to Fuel Hepatic Inflammation in Metabolic Dysfunction-Associated Steatotic Liver Disease
Source: Cells. 2026 Jan 16;15(2):165. doi: 10.3390/cells15020165 (PMC12839435; doi:10.3390/cells15020165)
Supplement: Supplementary file 1 [file cells-15-00165-s001.zip › cells-4020278-supplementary.pdf]

Western diet dampens T regulatory cell function to fuel hepatic inflammation in metabolic dysfunction-associated steatotic liver disease

Sudrishti Chaudhary[2,4]*, Ravi Rai[1,2]*, Pabitra B. Pal[2,4]*, Dana Tedesco[3], Daniel Rossmiller[4], Biki Gupta[2,4], Aatur D. Singhi[2,6], Satdarshan P. Monga[1,2,7], Arash Grakoui[3,5,8], Smita S. Iyer[4] and Reben Raeman[1,2#]

***Contributed equally**

TABLE OF CONTENTS

Supplementary methods ……………………………………………………….……..……2

Supp. Fig. 1 ……………………………………………………………………………..4

Supp. Fig. 2 ……………………………………………………………………………..5

Supp. Fig. 3 ……………………………………………………………………………..6-7

SUPPLEMENTARY METHODS

Histopathology. Formalin fixed, paraffin embedded liver tissue sections were stained with hematoxylin and eosin (H&E) and Sirius Red as described previously.[1] Images were captured using a Zeiss Light Microscope (Zeiss, Jena, Germany).

Immunofluorescence microscopy. Formalin fixed, paraffin embedded liver tissue sections were stained for immunofluorescence microscopy as described previously.[2] Sections were visualized using an Axioskop 2 plus laser confocal microscope. Following antibodies were used for human tissue, Foxp3 (cat. No PIPA585236, Thermo Fisher Scientific, Rockford, IL).

Serological analysis. Serum alanine aminotransferase (ALT) and aspartate aminotransferase (AST) concentrations were measured using an AST and ALT Activity Assay Kit (Sigma-Aldrich, St. Louis, MO) as described previously.[2]

Flow cytometric analysis. Flow cytometric analysis was performed on hepatic lymphocytes as described previously.[2] Briefly, livers perfused with 1X PBS were digested with 2 mg/mL type IV collagenase (Worthington, NY) to obtain single cell suspension. Lymphocytes were then enriched by Percoll gradient centrifugation, stained with fluorochrome-conjugated antibodies and fixable viability stain (BD Biosciences, San Jose, CA) and acquired on a Cytek Aurora Spectral Cytometer (Cytek Biosciences, Bethesda, MD) equipped with five lasers. The data obtained were analyzed using FlowJo software v X.10 (Tree Star, Inc., Ashland, OR). Fluorochrome-conjugated antibodies against CD3 (clone 17A2, PE-Cy7), CD4 (clone RM4-5, PerCP-Cy5.5), CD8 (clone 53-6.7, eFluor 780), CD44 (clone IM7, Pacific Blue), and Ly6C (clone AL-21, PE-CF594) were purchased from BD Biosciences (San Jose, CA). Foxp3 (clone FJK-16s, APC), Ki67 (clone SolA15, Qdot 800), Ly6G (clone RB6-8C5, Pacific Blue), CD11b (clone M1/70, eFluor 780), and Live/Dead Aqua (AmCyan) were purchased from Thermo Fisher Scientific

(Rockford, IL). PD-1 (clone RMP1-30, PE) and F4/80 (clone BM8, PE-Cy7) were purchased from BioLegend (San Diego, CA). Separate panels were used for innate and T cell phenotyping.

Quantitative real-time PCR. Isolation of total RNA from tissue, synthesizing complementary DNA (cDNA) from RNA and qRT-PCRs were carried out as described previously.[1] Data were normalized to the housekeeping gene 18S rRNA and presented as fold change in gene expression relative to controls.

REFERENCES.

1. Rahman K, Desai C, Iyer SS, et al. Loss of Junctional Adhesion Molecule A Promotes Severe Steatohepatitis in Mice on a Diet High in Saturated Fat, Fructose, and Cholesterol. Gastroenterology 2016.
2. Rai RP, Liu Y, Iyer SS, et al. Blocking integrin alpha4beta7-mediated CD4 T cell recruitment to the intestine and liver protects mice from western diet-induced non-alcoholic steatohepatitis. J Hepatol 2020.

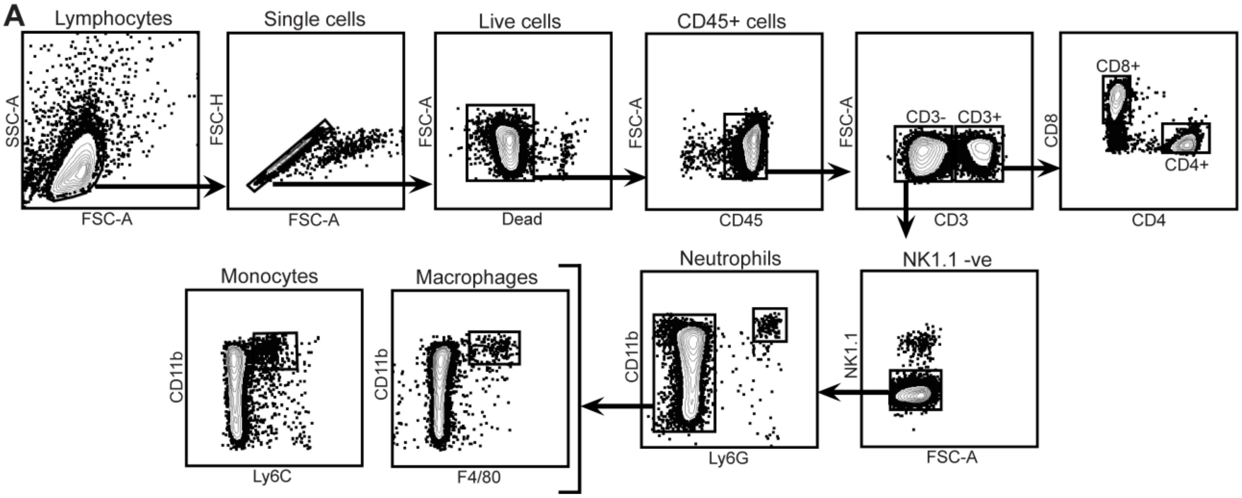

Supp. Fig. 1. Gating strategy used to identify intrahepatic lymphoid and myeloid cell subsets.

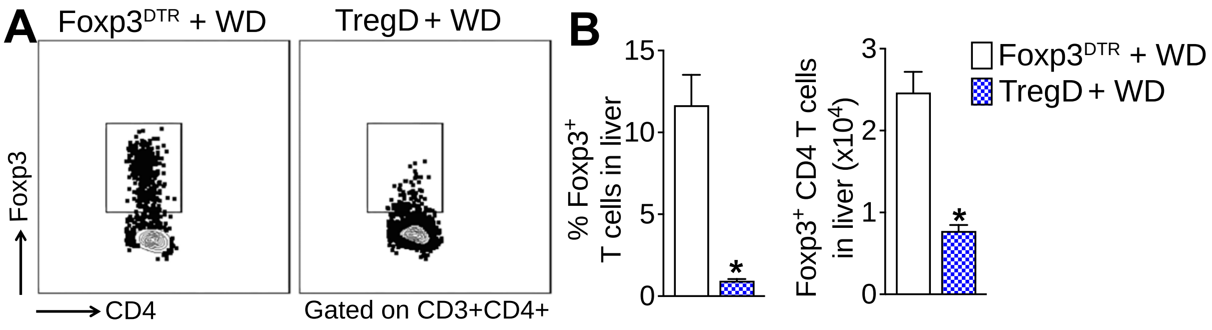

Supp. Fig. 2. Depletion of Foxp3+ regulatory T cells in Foxp3^{DTR} mice. (**A**) Representative flow plots show percent of Foxp3+ regulatory T cells in the liver. (**B**) Bar graphs show percent and total number of Foxp3+ regulatory T cells in the liver. A cohort of Foxp3^{DTR} mice fed a WD for sixteen-weeks were randomized to receive weekly intraperitoneal injections of diphtheria toxin (DT, TregΔ) or saline (controls) for four weeks starting at week twelve. Data are representative of 3 independent experiments (n = 5 mice per group). Data are presented as mean ± SEM. Asterisks indicate significant differences ($p < 0.05$) between TregΔ and control mice.

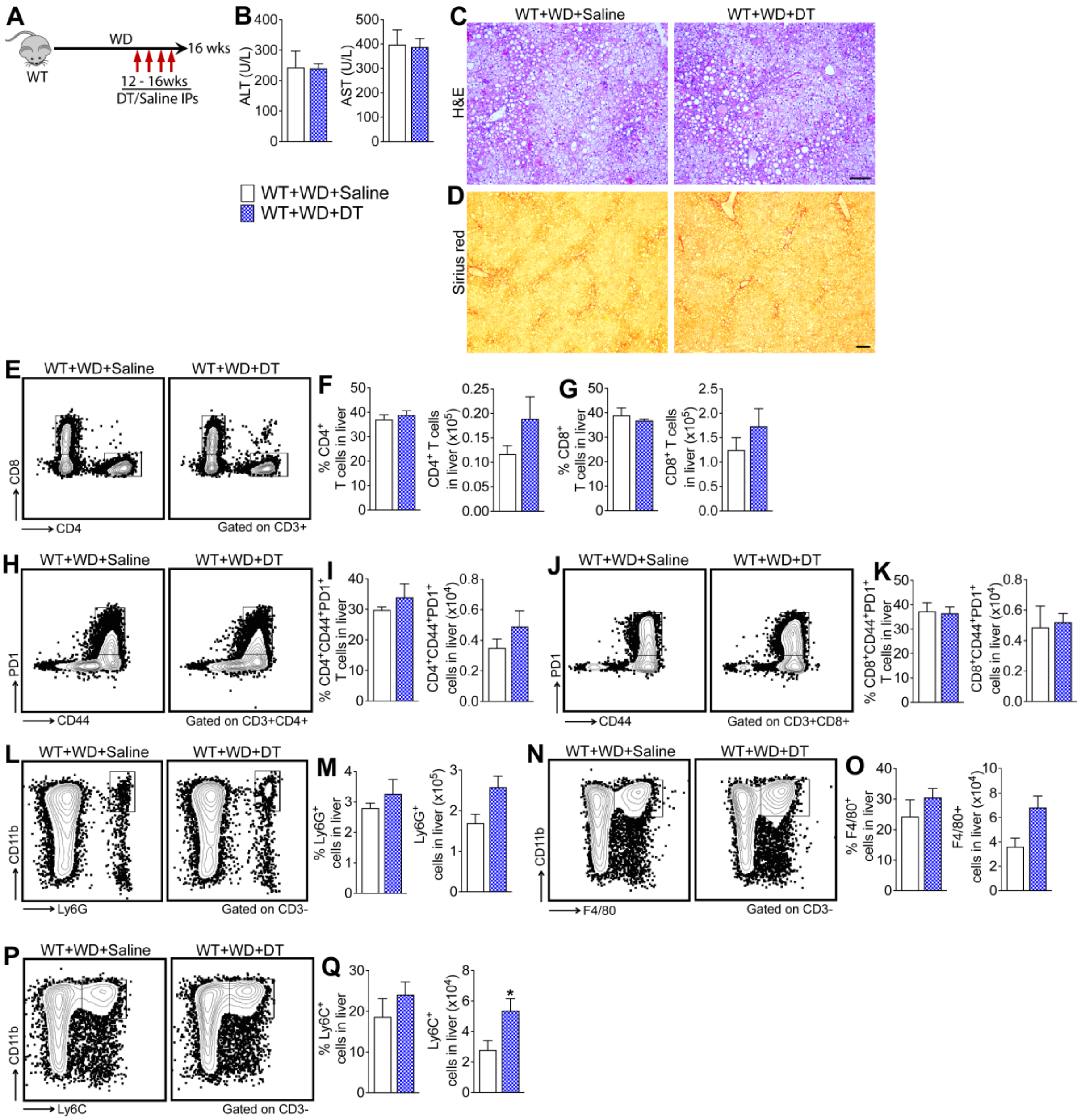

Supp. Fig. 3. No differences in hepatic inflammation and fibrosis in WD-fed DTR-negative mice treated with DT. (**A**) Schematic of study design. A cohort of DTR-negative (WT) mice fed a WD for sixteen-weeks were randomized to receive weekly intraperitoneal injections of diphtheria toxin (DT) or saline (controls) for four weeks starting at week twelve. (**B**) Serum AST and ALT levels. (**C**) Representative photomicrographs of Hematoxylin and Eosin (H&E) stained liver tissue

sections. (**D**) Representative photomicrographs of Sirius Red-stained liver tissue sections. (**E-G**) Representative flow plots show percent of intrahepatic CD4 and CD8 T cells. Bar graphs show percent and total number of intrahepatic (**F**) CD4 and (**G**) CD8 T cells. (**H**) Representative flow plots show percent of intrahepatic PD-1+CD44+ CD4 T cells and (**I**) bar graphs show percent and total number of PD-1+CD44+ CD4 T cells. (**J**) Representative flow plots show percent of intrahepatic PD-1+CD44+ CD8 T cells and (**K**) bar graphs show percent and total number of intrahepatic PD-1+CD44+ CD8 T cells. (**L**) Representative flow plots show percent of intrahepatic neutrophils (CD11b+Ly6G+ cells) and (**M**) bar graphs show percent and total number of intrahepatic neutrophils. (**N**) Representative flow plots show percent of intrahepatic macrophages (CD11b+F4/80+ cells) and (**O**) bar graphs show percent and total number of intrahepatic macrophages. (**P**) Representative flow plots show percent of intrahepatic monocytes (CD11b+Ly6C+ cells) and (**Q**) bar graphs show percent and total numbers of intrahepatic monocytes. Data are representative of 3 independent experiments ($n = 5$ mice per group). Data are presented as mean ± SEM. Asterisks indicate significant differences ($p < 0.05$) between DT-treated and Saline-treated mice.
